# Supplementary material for: Microscopic origins of the terahertz carrier relaxation and cooling dynamics in graphene
Source: Nat Commun. 2016 May 25;7:11617. doi: 10.1038/ncomms11617 (PMC4894949; doi:10.1038/ncomms11617)
Supplement: Supplementary Information — Supplementary Figures 1-6, Supplementary Table 1, Supplementary Notes 1-6 and Supplementary References [file ncomms11617-s1.pdf]

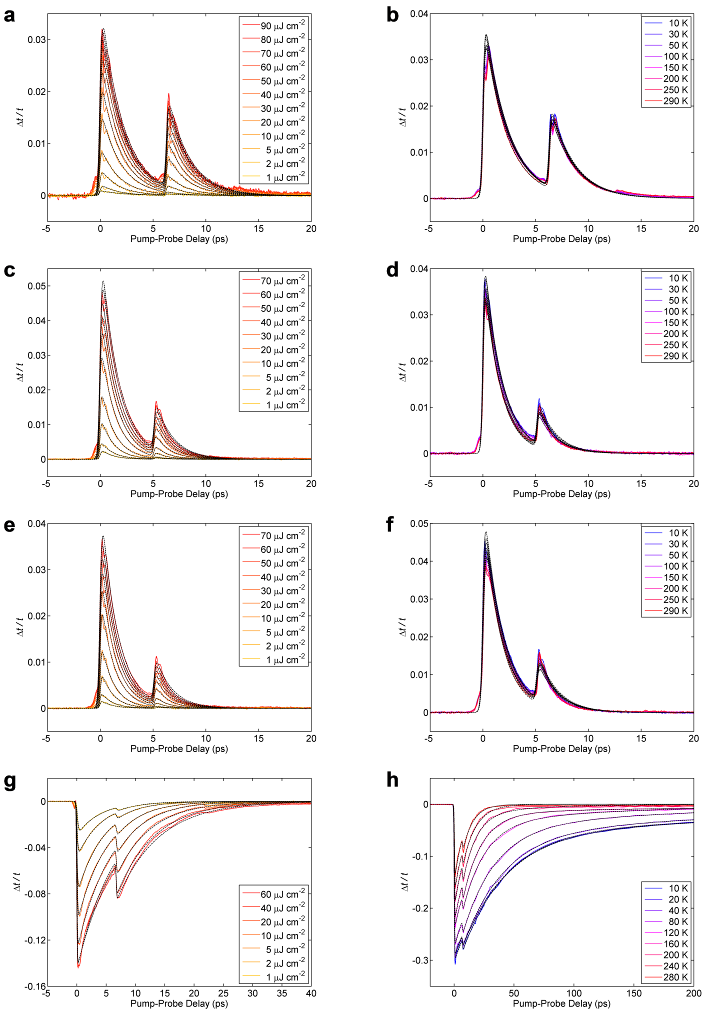

Supplementary Figure 1. **THz carrier dynamics in various types of graphene.** a-b, Experimental differential THz transmission, $\Delta t/t$, as a function of pump-probe delay recorded at a substrate temperature of 300 K for a few different pump fluences (a) and at a pump fluence of 60.0 μJ cm^{-2} for a few different substrate temperatures (b) for a highly doped MEG sample with $\sim$ 3 layers. c-d, The same measurements as in (a-b), but for a highly doped sCVDG sample with $\sim$ 1 layer. e-f, The same measurements as in (a-b), but for a highly doped pCVDG sample with $\sim$ 1 layer. The THz carrier dynamics in all highly doped graphene samples follow a fast mono-exponential relaxation at all substrate temperatures and all pump fluences (dashed black lines). g-h, Experimental differential THz transmission, $\Delta t/t$, as a function of pump-probe delay recorded at a substrate temperature of 300 K for a few different pump fluences (g) and at a pump fluence of 23.4 μJ cm^{-2} for a few different substrate temperatures (h) for a lightly doped MEG sample with $\sim$ 63 layers. The THz carrier dynamics in all lightly doped graphene samples evolve from a faster mono-exponential relaxation at room temperature to a slower bi-exponential relaxation at cryogenic temperatures (dashed black lines).

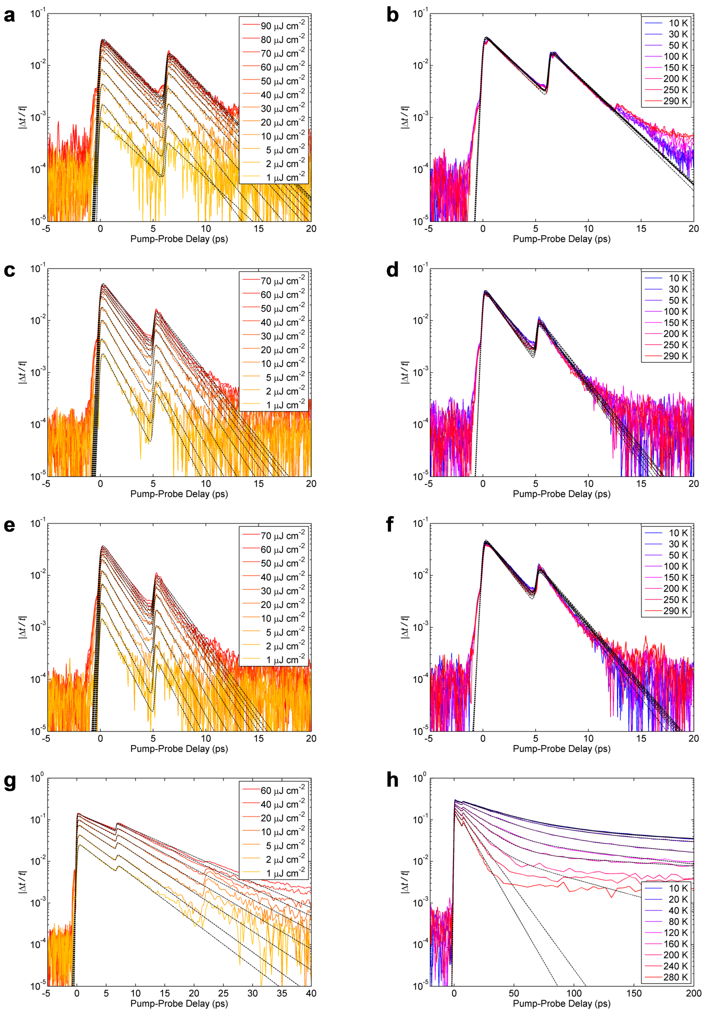

Supplementary Figure 2. **THz carrier dynamics in various types of graphene.** a-b, Experimental differential THz transmission, $\Delta t/t$, as a function of pump-probe delay recorded at a substrate temperature of 300 K for a few different pump fluences (a) and at a pump fluence of 60.0 μJ cm^{-2} for a few different substrate temperatures (b) for a highly doped MEG sample with ~ 3 layers. c-d, The same measurements as in (a-b), but for a highly doped sCVDG sample with ~ 1 layer. e-f, The same measurements as in (a-b), but for a highly doped pCVDG sample with ~ 1 layer. The THz carrier dynamics in all highly doped graphene samples follow a fast mono-exponential relaxation at all substrate temperatures and all pump fluences (dashed black lines). g-h, Experimental differential THz transmission, $\Delta t/t$, as a function of pump-probe delay recorded at a substrate temperature of 300 K for a few different pump fluences (g) and at a pump fluence of 23.4 μJ cm^{-2} for a few different substrate temperatures (h) for a lightly doped MEG sample with ~ 63 layers. The THz carrier dynamics in all lightly doped graphene samples evolve from a faster mono-exponential relaxation at room temperature to a slower bi-exponential relaxation at cryogenic temperatures (dashed black lines).

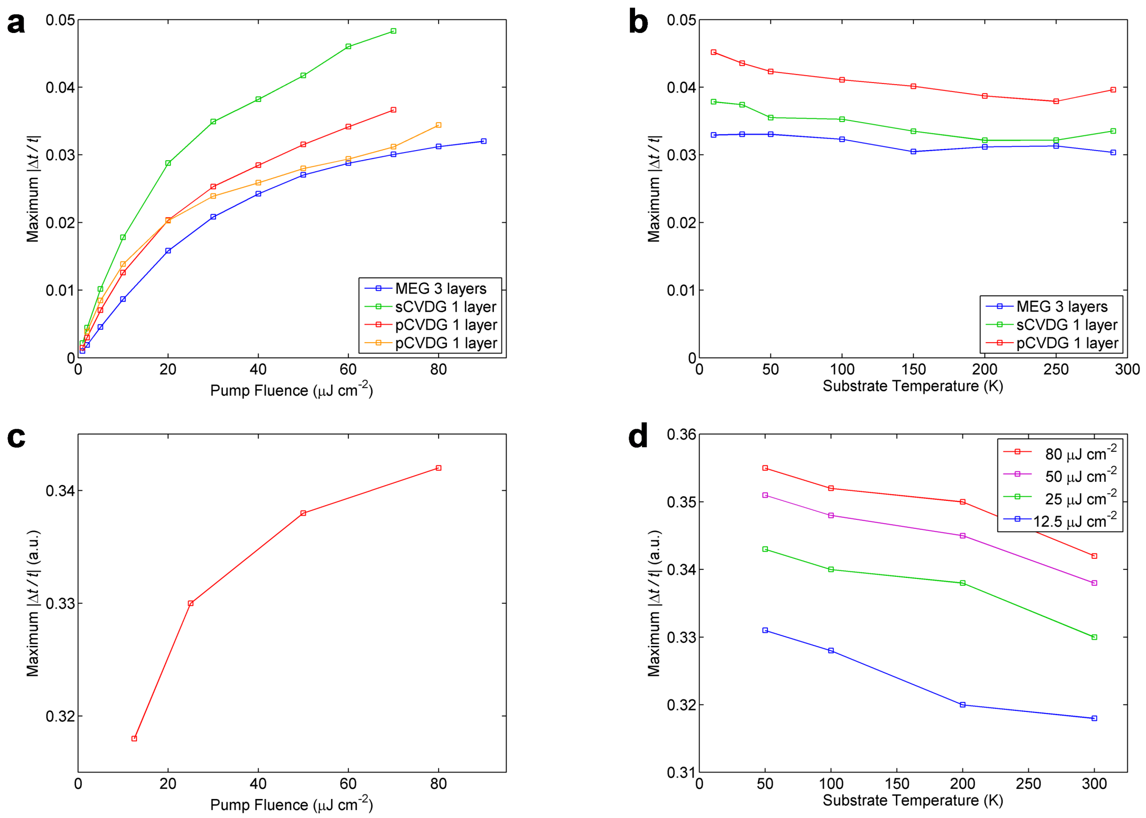

Supplementary Figure 3. **Maximum signal in the THz carrier dynamics in graphene with high doping density.** a-b, Maximum absolute value of experimental differential THz transmission, maximum $|\Delta t/t|$, as a function of pump fluence at a substrate temperature of 300 K (a) and as a function of substrate temperature at a pump fluence of 60.0 μJ cm^{-2} (b) for highly doped MEG, sCVDG and pCVDG samples. c-d, Maximum absolute value of theoretical differential THz transmission, maximum $|\Delta t/t|$, as a function of pump fluence at a substrate temperature of 300 K (c) and as a function of substrate temperature for a few different pump fluences (d) for disorder-free highly doped graphene ($|\varepsilon_{\rm F}| = 300$ meV). Experiment and theory are in excellent agreement under all conditions.

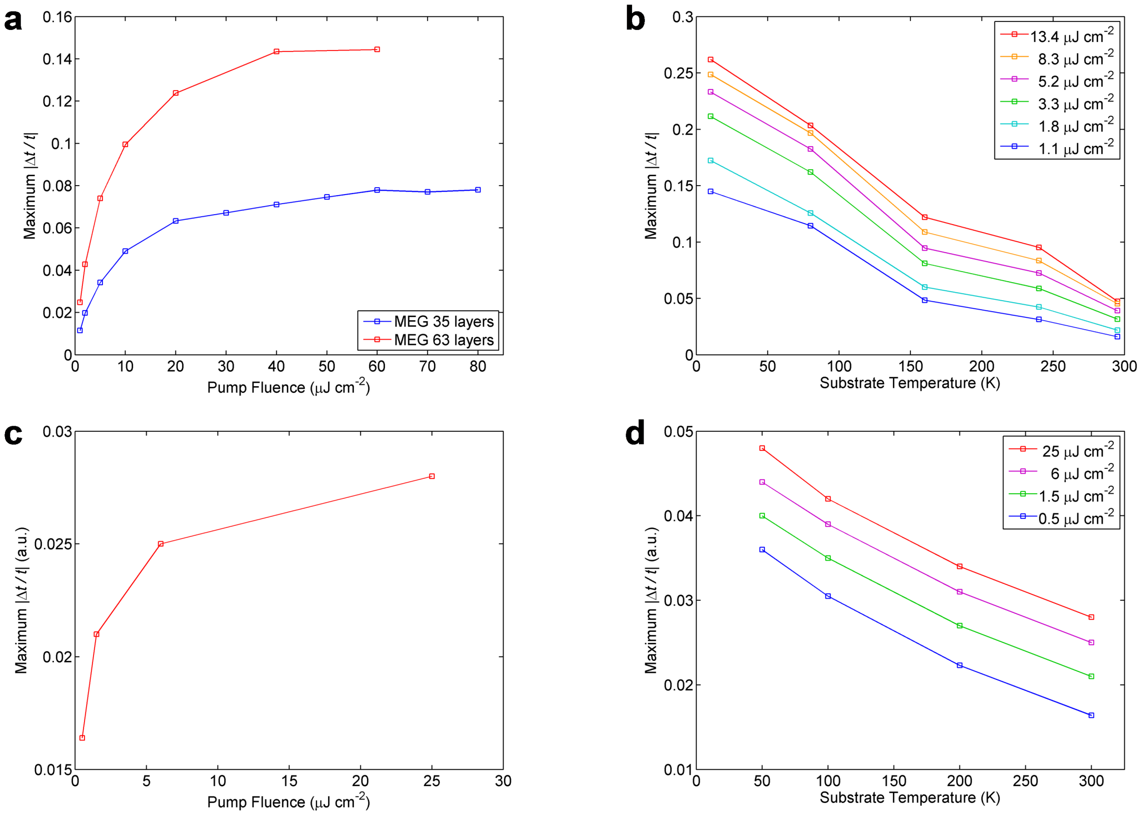

Supplementary Figure 4. **Maximum signal in the THz carrier dynamics in graphene with low doping density.** a-b, Maximum absolute value of experimental differential THz transmission, maximum $|\Delta t/t|$, as a function of pump fluence at a substrate temperature of 300 K (a) and as a function of substrate temperature for a few different pump fluences (b) for lightly doped MEG samples. c-d, Maximum absolute value of theoretical differential THz transmission, maximum $|\Delta t/t|$, as a function of pump fluence at a substrate temperature of 300 K (c) and as a function of substrate temperature for a few different pump fluences (d) for disorder-free undoped graphene ($|\varepsilon_{\rm F}| = 0$ meV). Experiment and theory are in excellent agreement under all conditions.

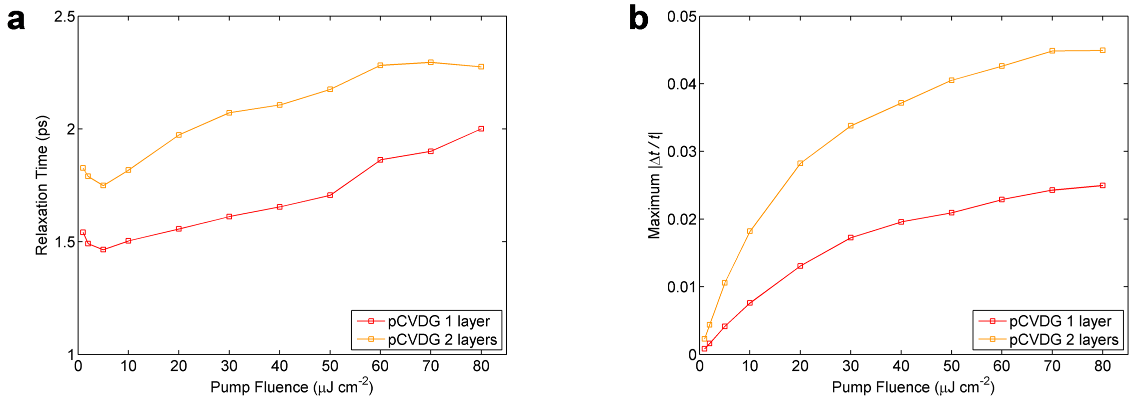

Supplementary Figure 5. **THz carrier dynamics in mono- and bi-layer pCVDG.** a, Carrier relaxation times extracted from fits to experimental differential THz transmission, $\Delta t/t$, as a function of pump fluence at a substrate temperature of 300 K for highly doped mono- and bi-layer pCVDG samples. b, Maximum absolute value of experimental differential THz transmission, maximum $|\Delta t/t|$, as a function of pump fluence at a substrate temperature of 300 K for highly doped mono- and bi-layer pCVDG samples.

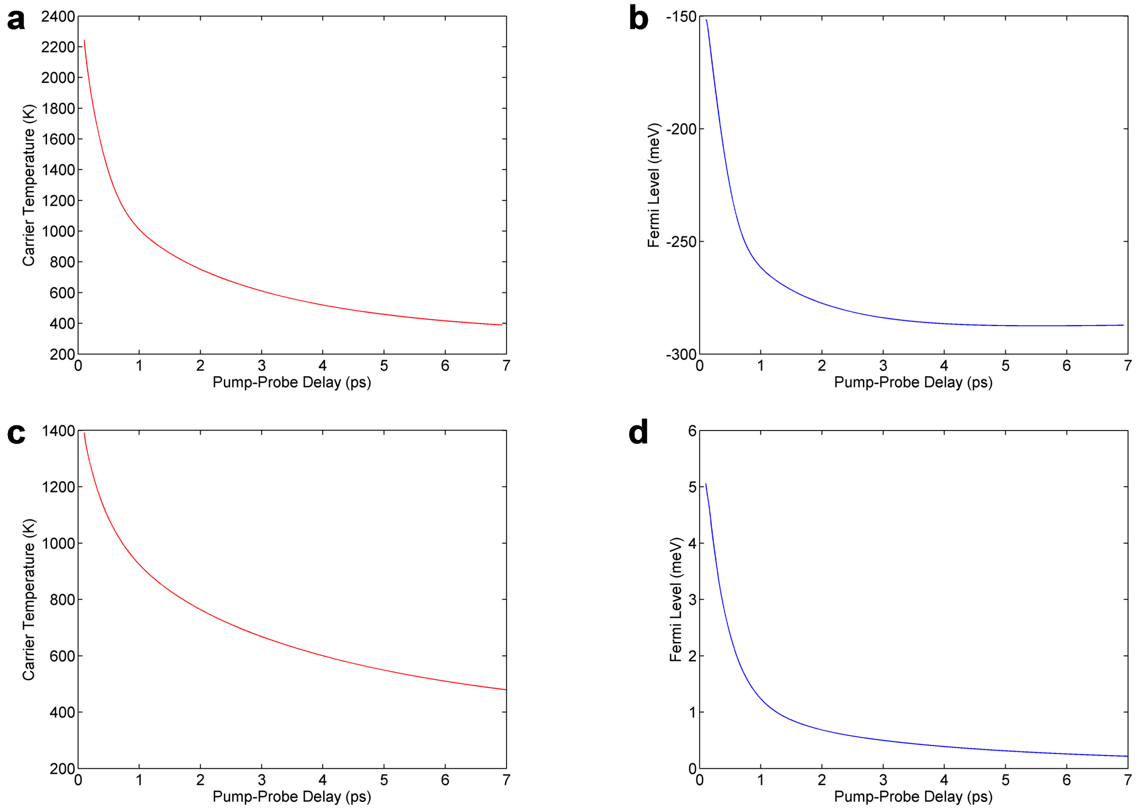

Supplementary Figure 6. **Carrier temperature and Fermi level dynamics in the microscopic theory.** a-b, Carrier temperature dynamics (a) and Fermi level dynamics (b) calculated within the microscopic theory at a substrate temperature of 300 K and a pump fluence of 12.5 μJ cm^{-2} for disorder-free highly doped graphene ($|\varepsilon_{\rm F}| = 300$ meV). c-d, Carrier temperature dynamics (c) and Fermi level dynamics (d) calculated within the microscopic theory at a substrate temperature of 300 K and a pump fluence of 1.5 μJ cm^{-2} for disorder-free undoped graphene ($|\varepsilon_{\rm F}| = 0$ meV).

SUPPLEMENTARY TABLES

Supplementary Table 1. **THz carrier dynamics in various types of graphene.**

Graphene sample	Absolute value of Fermi level (meV)	Differential THz transmission	Relaxation time (ps)	Substrate temperature dependence
Multilayer epitaxial graphene (MEG)	~10 (quasi-neutral)	negative	~4-40 (short)<br>~50-500 (long)	strong dependence
	~100-400 (n-doped)	positive	~1-3	no dependence
Single-crystal CVD graphene (sCVDG)	~200-400 (p-doped)	positive	~1-3	no dependence
Polycrystalline CVD graphene (pCVDG)	~200-400 (p-doped)	positive	~1-3	no dependence

SUPPLEMENTARY NOTES

Supplementary Note 1. Hot-carrier relaxation and cooling dynamics in graphene with various doping densities

Here, we present additional detailed description of the hot-carrier relaxation and cooling dynamics in graphene with various doping densities. A schematic illustration of the hot-carrier dynamics in graphene following ultrafast photoexcitation appears in Figure 1 in the main text, which shows how they depend critically on the Fermi level. It is important to distinguish between carrier thermalization via carrier-carrier scattering, which only redistributes the deposited energy within the electron gas, and carrier cooling via carrier-phonon scattering, which takes energy from the electron gas into the phonon system (that is, the lattice). Figure 1a-b in the main text show the hot-carrier dynamics in highly doped graphene. Initially, the ultrafast optical pump pulse injects high-energy non-equilibrium electrons in the conduction band and holes in the valence band. Strong intraband and interband carrier-carrier scattering lead to ultrafast carrier relaxation and thermalization which establish a single uniform hot-carrier Fermi-Dirac distribution within $\sim 100 - 200$ fs after photoexcitation [1–3]. Hot-carrier cooling proceeds further via optical phonon emission on a timescale of a few picoseconds. It is essentially facilitated by these extraordinarily efficient Coulomb interactions which continuously rethermalize the hot-carrier distribution and replenish the carriers at high energies which emit optical phonons.

The situation in undoped (very lightly doped) graphene is very different, as shown in Figure 1c in the main text. Immediately after photoexcitation, the phase space for impact ionization is large, while Auger recombination is inhibited, which leads to significant carrier multiplication in the conduction band up to a moderate excitation regime [4, 5]. Again, the strong intraband and interband carrier-carrier scattering establish a single uniform hot-carrier Fermi-Dirac distribution on an ultrafast timescale [1–3] followed by hot-carrier cooling via optical phonon emission. At later times, the small phase space near the Dirac point strongly reduces the efficiency of carrier rethermalization via the Coulomb interactions, which slows down optical phonon emission and hot-carrier cooling which last on a timescale exceeding hundreds of picoseconds at cryogenic temperatures.

The hot-carrier relaxation and cooling processes illustrated in Figure 1 in the main text

are directly accessible via the dynamic response of the system to a THz probe pulse. Qualitatively, for all types of graphene having high doping density, we observe in both experiment and theory a fast and substrate-temperature-independent THz carrier dynamics. On the other hand, for graphene having very low doping density, we observe a comparatively slower and strongly substrate-temperature-dependent THz carrier dynamics. A second feature of our experiments is that we find that graphene samples with very different degrees of disorder show essentially the same hot-carrier relaxation times; this suggests that disorder-assisted electron-phonon (supercollision) scattering might not play a crucial role in the hot-carrier dynamics as has been proposed in previous literature [6–8]. We show that the dependence of the dynamic THz response on Fermi level, substrate temperature and initial carrier temperature, and its independence of disorder, can be quantitatively understood in the microscopic theory.

Supplementary Note 2. THz carrier dynamics in various types of graphene

Here, we present additional experimental data on the THz carrier dynamics in all types of graphene samples that we have studied to further illustrate the conclusions in the main text. Supplementary Figures 1 and 2 show the differential THz transmission signal at the peak of the THz probe pulse normalized to the THz transmission without photoexcitation, $\Delta t/t$, as a function of pump-probe delay, for variable pump fluence and for variable substrate temperature, respectively, for a highly doped MEG sample (a-b), a highly doped sCVDG sample (c-d), a highly doped pCVDG sample (e-f) and a lightly doped MEG sample (g-h). Based on extensive measurements on many graphene samples under various conditions, we observe that the differential THz transmission is positive for all highly doped graphene samples, which corresponds to a pump-induced increase of the THz transmission or a decrease of the THz absorption, and negative for all lightly doped graphene samples, which corresponds to a pump-induced decrease of the THz transmission or an increase of the THz absorption, under all experimental conditions. In addition, the differential THz transmission of all highly doped graphene samples follows closely a fast and substrate-temperature-independent mono-exponential relaxation (dashed black lines in Supplementary Figures 1 and 2) with relaxation times on the order of a few picoseconds that vary from sample to sample within $\sim 20-30\%$. These THz carrier dynamics are very well explained by the microscopic theory of carrier-carrier and carrier-phonon interactions in a disorder-free graphene system. On the other hand, the differential THz transmission of all lightly doped graphene samples follows closely a comparatively slower and strongly substrate-temperature-dependent bi-exponential relaxation (dashed black lines in Supplementary Figures 1 and 2) with a short and a long relaxation component governed by two distinct cooling mechanisms. The short relaxation times are on the order of a few to a few tens of picoseconds varying similarly from sample to sample within $\sim 20-30\%$ and are also very well explained by the microscopic theory. The long relaxation times extend up to on the order of hundreds of picoseconds monotonically increasing with the number of epitaxial graphene layers and are attributed to a unique cooling mechanism enabled by interlayer energy transfer via screened Coulomb interactions [9]. Supplementary Table 1 summarizes the key characteristics of the THz carrier dynamics in the various graphene samples.

Supplementary Note 3. Maximum signal in the THz carrier dynamics

Here, we present additional evidence to further illustrate the excellent agreement between the experimental data and the theoretical calculations of the THz carrier dynamics in all types of graphene samples that we have studied. Supplementary Figure 3a-b show the maximum absolute value of the differential THz transmission at the peak of the THz probe pulse, maximum $|\Delta t/t|$, as a function of pump fluence and substrate temperature, respectively, for highly doped MEG, sCVDG and pCVDG samples, while Supplementary Figure 3c-d show the results obtained within the microscopic theory for disorder-free highly doped graphene ($|\varepsilon_{\mathrm{F}}| = 300$ meV) under similar experimental conditions. We observe that the theory captures very well the saturation of the maximum $|\Delta t/t|$ signal with the pump fluence and its invariance with the substrate temperature for highly doped graphene samples. Supplementary Figure 4a-b show the maximum absolute value of the differential THz transmission at the peak of the THz probe pulse, maximum $|\Delta t/t|$, as a function of pump fluence and substrate temperature, respectively, for lightly doped MEG samples, while Supplementary Figure 4c-d show the results obtained within the microscopic theory for disorder-free undoped graphene ($|\varepsilon_{\mathrm{F}}| = 0$ meV) under similar experimental conditions. Similarly, we observe that the theory captures very well the saturation of the maximum $|\Delta t/t|$ signal with the pump fluence and its decrease with increasing the substrate temperature. We can also point out that the maximum $|\Delta t/t|$ signal scales roughly linearly with the number of graphene layers, when the individual layers have similar Fermi levels and degrees of disorder, and hence similar intraband THz conductivities. This is because the intraband THz conductivities of the individual layers simply add up to first order, since they are electronically decoupled.

Supplementary Note 4. THz carrier dynamics in mono- and bi-layer pCVDG

Here, we present additional experimental data on the THz carrier dynamics in pCVDG samples by varying the number of graphene layers and the underlying substrate to further illustrate the conclusions in the main text. Supplementary Figure 5a-b show the extracted carrier relaxation times and the maximum absolute value of the differential THz transmission at the peak of the THz probe pulse, maximum $|\Delta t/t|$, as a function of pump fluence for a highly doped mono- and bi-layer pCVDG samples transferred on an amorphous polyethylene (TOPAS cyclic olefin copolymer, TOPAS Advanced Polymers) substrate. We observe that the relaxation times of both highly doped mono- and bi-layer pCVDG samples are very similar in value and also very similar to all other highly doped graphene samples with sample-to-sample variation within $\sim 20 - 30\%$. We also observe that the maximum $|\Delta t/t|$ signal scales roughly linearly with the number of graphene layers, when the individual layers have similar Fermi levels and degrees of disorder, and hence similar intraband THz conductivities. This is because the intraband THz conductivities of the individual layers simply add up to first order. We note that based on all our extensive experimental data and microscopic theory calculations we can conclude that the THz carrier dynamics in all highly doped graphene samples are very weakly dependent on the graphene fabrication method, the degree of disorder, the number of graphene layers and their stacking orientation, the substrate temperature, and the type of underlying substrate, but more strongly dependent on the pump fluence due to the re-absorption of hot optical phonons generated during the initial carrier thermalization as explained in detail in the main text.

Supplementary Note 5. Carrier temperature and Fermi level dynamics in the microscopic theory

Here, we present additional detailed description of the carrier temperature and Fermi level dynamics in the microscopic theory. The balance between carrier density and energy density of the carrier system uniquely defines both the transient carrier temperature $T(t)$ and the transient Fermi level $\varepsilon_\mathrm{F}(t)$. Apart from phonon-induced carrier recombination and energy dissipation, carrier-carrier scattering affects the carrier density via Auger processes. Thus, a microscopic description of the carrier relaxation is essential to capture the dynamics of carrier temperature and Fermi level accurately. Based on numerical solution of the full graphene Bloch equations of the pump-induced carrier dynamics, we determine the temporal evolution of both $T(t)$ and $\varepsilon_\mathrm{F}(t)$ by fitting the microscopic carrier distribution at each time step. The results are shown in Supplementary Figure 6a-b for disorder-free highly doped graphene ($|\varepsilon_\mathrm{F}| = 300$ meV) and in Supplementary Figure 6c-d for undoped graphene ($|\varepsilon_\mathrm{F}| = 0$ meV). During the initial $\sim 100 - 200$ fs after the pump pulse carrier thermalization forms a single uniform Fermi-Dirac distribution as well as orientational relaxation takes place. Afterwards, carrier temperature and Fermi level are well defined. We find carrier temperatures in the range of $\sim 1000 - 2000$ K, which cool on a picosecond timescale for both highly doped and undoped graphene, as shown in Supplementary Figure 6a and c. The Fermi level for highly doped graphene, shown in Supplementary Figure 6b, drastically changes due to the pump pulse. We find a reduction from -300 meV to temporarily -150 meV. The subsequent recovery takes place on the same picosecond timescale as carrier cooling. This dynamics of the transient Fermi level is crucial for the understanding of the THz carrier dynamics, in particular for the sign of the differential THz transmission, see also Figure 6a in the main text. The amplitude of the Fermi level for undoped graphene, shown in Supplementary Figure 6d, is negligible and in the range of the accuracy of its determination scheme.

Supplementary Note 6. Comparison between the microscopic theory and the standard Drude model

Here, we present additional detailed description of the comparison between the microscopic theory and the standard Drude model. In order to obtain additional insight into the physics governing the THz carrier dynamics, we consider a simplified model by assuming a constant time- and momentum-independent scattering kernel for the probe-induced carrier dynamics; we show in Methods that this approach yields a standard Drude model. The transient carrier temperature $T(t)$ and Fermi level shift $\varepsilon_{\mathrm{F}}(t)$ are formed after the thermalization of the pump-induced non-equilibrium carrier population. The Drude-like $\Delta t/t$ signal calculated for disorder-free highly doped graphene ($|\varepsilon_{\mathrm{F}}| = 300$ meV) is mapped in Figure 6a in the main text. It is apparent (see the dashed black line in Figure 6a in the main text) that if the effect of the optical pump was only to heat the carriers without changing the Fermi level, then a *negative* sign of the $\Delta t/t$ signal would always be observed. We conclude that, for a constant scattering rate Γ, carrier heating alone cannot explain the positive sign of the $\Delta t/t$ signal observed in highly doped graphene.

Figure 6a in the main text also illustrates the pump-induced temporal evolution of $T(t)$ and $\varepsilon_{\mathrm{F}}(t)$ obtained by solving the full graphene Bloch equations within this approximation for low (12.5 μJ cm^{-2} (red line)) and high (80 μJ cm^{-2} (blue line)) photoexcitation. Interestingly, we find not only carrier heating, but also a transient decrease of the Fermi level which reduces the THz absorption. Obviously, the pump-induced shift of the Fermi level outweighs the impact of carrier heating. As a consequence, we find a *positive* sign of the $\Delta t/t$ signal for the lower pump fluence (red line) at zero time delay and for the higher pump fluence (blue line) just after 0.8 ps. Thus, the positive $\Delta t/t$ signal for highly doped graphene observed in the measurements can be explained to a large extent by a standard Drude model with constant Γ, provided that the carrier heating and cooling dynamics and the Fermi level dynamics are described correctly. Previously, the observation of a positive $\Delta t/t$ signal in doped graphene has been attributed phenomenologically to a pump-induced increase of the carrier scattering rate, and a corresponding decrease of the THz conductivity within a Drude model [10–12], which is essentially a metal-like behavior, although the mechanism responsible for the increase in the carrier scattering rate could not be positively identified. Here, we find that the positive $\Delta t/t$ signal can be partly explained within a simple Drude picture

as a consequence of the Fermi level shift. However, the simple Drude model is not sufficient to explain the behavior at higher pump fluence, for which the short time dynamics would exhibit a negative $\Delta t/t$ signal, unless other phenomenological parameters such as a carrier heating efficiency [13, 14] or a non-monotonic carrier-temperature-dependent Drude weight [15] are included.

A complete understanding is obtained by applying the full microscopic formalism including the explicitly time- and momentum-dependent scattering rates $\Gamma^0_{\lambda\mathbf{k}}(t)$ of the probe-induced carrier dynamics. The results are shown in Figure 2c-d in the main text, exhibiting excellent agreement with the experiment under all conditions. Specifically, we observe, in both experiment and theory, an overall positive $\Delta t/t$ signal at all time delays and all pump fluences. Thus, the microscopic model for the pump-induced carrier scattering is essential to capture the very initial THz carrier dynamics correctly. In the regime of high fluence and short time, even if carrier thermalization is complete, a constant carrier scattering rate approximation cannot be trusted. In particular, we find that in the strong excitation regime efficient time-dependent Coulomb scattering provides the main contribution. All results presented in the comparisons with experiment are obtained from the full microscopic formalism beyond the standard Drude model.

SUPPLEMENTARY REFERENCES

---

[1] M. Breusing, S. Kuehn, T. Winzer, E. Malić, F. Milde, N. Severin, J. P. Rabe, C. Ropers, A. Knorr and T. Elsaesser, Ultrafast nonequilibrium carrier dynamics in a single graphene layer, *Phys. Rev. B* **83**, 153410 (2011).

[2] D. Brida, A.Tomadin, C. Manzoni C., Y. J. Kim, A. Lombardo A., S. Milana, R. R. Nair, K. S. Novoselov, A. C. Ferrari, G. Cerullo and M. Polini, Ultrafast collinear scattering and carrier multiplication in graphene, *Nat. Commun.* **4**, 1987 (2013).

[3] I. Gierz, J. C. Petersen, M. Mitrano, C. Cacho, I. C. E. Turcu, E. Springate, A. Stöhr, A. Köhler Axel, U. Starke and A. Cavalleri, Snapshots of non-equilibrium Dirac carrier distributions in graphene, *Nat. Mater.* **12**, 1119-1124 (2013).

[4] T. Winzer, A. Knorr and E. Malic, Carrier Multiplication in Graphene, *Nano Lett.* **10**, 4839-4843 (2010).

[5] T. Plötzing, T. Winzer, E. Malic, D. Neumaier, A. Knorr and H. Kurz, Experimental Verification of Carrier Multiplication in Graphene, *Nano Lett.* **14**, 5371-5375 (2014).

[6] J. C. W. Song, M. Y. Reizer and L. S. Levitov, Disorder-assisted electron-phonon scattering and cooling pathways in graphene, *Phys. Rev. Lett.* **109**, 106602 (2012).

[7] M. W. Graham, S.-F. Shi, D. C. Ralph, J. Park and P. L. McEuen, Photocurrent measurements of supercollision cooling in graphene, *Nat. Phys.* **9**, 103-108 (2013).

[8] A. C. Betz, S. H. Jhang, E. Pallecchi, R. Ferreira, G. Feve, J.-M. Berroir and B. Placais, Supercollision cooling in undoped graphene, *Nat. Phys.* **9**, 109-112 (2013).

[9] M. T. Mihnev, J. R. Tolsma, C. J. Divin, D. Sun, R. Asgari, M. Polini, C. Berger, W. A. de Heer, A. H. MacDonald and T. B. Norris, Electronic cooling via interlayer Coulomb coupling in multilayer epitaxial graphene, *Nat. Commun.* **6**, 8105 (2015).

[10] G. Jnawali, Y. Rao, H. Yan and T. F. Heinz, Observation of a transient decrease in terahertz conductivity of single-layer graphene induced by ultrafast optical excitation, *Nano Lett.* **13**, 524-530 (2013).

[11] S.-F. Shi, T.-T. Tang, B. Zeng, L. Ju, Q. Zhou, A. Zettl and F. Wang, Controlling graphene ultrafast hot carrier response from metal-like to semiconductor-like by electrostatic gating, *Nano Lett.* **14**, 1578-1582 (2014).

[12] A. J. Frenzel, C. H. Lui, W. Fang, N. L. Nair, P. K. Herring, P. Jarillo-Herrero, J. Kong and N. Gedik, Observation of suppressed terahertz absorption in photoexcited graphene, *Appl. Phys. Lett.* **102**, 113111 (2013).

[13] K. J. Tielrooij, J. C. W. Song, S. A. Jensen, A. Centeno, A. Pesquera, A. Zurutuza Elorza, M. Bonn, L. S. Levitov and F. H. L. Koppens, Photoexcitation cascade and multiple hot-carrier generation in graphene, *Nat. Phys.* **9**, 248-252 (2013).

[14] S. A. Jensen, Z. Mics, I. Ivanov, H. S. Varol, D. Turchinovich, F. H. L. Koppens, M. Bonn and K. J. Tielrooij, Competing ultrafast energy relaxation pathways in photoexcited graphene, *Nano Lett.* **14**, 5839-5845 (2014).

[15] A. J. Frenzel, C. H. Lui, Y. C. Shin, J. Kong and N. Gedik, Semiconducting-to-metallic photoconductivity crossover and temperature-dependent Drude weight in graphene, *Phys. Rev. Lett.* **113**, 056602 (2014).
